# Crystal Analyzer Based Multispectral Microtomography Using CCD-Sensor

**DOI:** 10.3390/s23146389

**Published:** 2023-07-14

**Authors:** Maxim Grigoriev, Denis Zolotov, Anastasia Ingacheva, Alexey Buzmakov, Irina Dyachkova, Victor Asadchikov, Marina Chukalina

**Affiliations:** 1 Institute of Microelectronics Technology and High Purity Materials RAS, Osipyan Str., 6, 142432 Chernogolovka, Russia; grimax@iptm.ru; 2 FSRC “Crystallography and Photonics” RAS, Leninskiy Prospekt 59, 119333 Moscow, Russia; zolotovden@crys.ras.ru (D.Z.); buzmakov@crys.ras.ru (A.B.); asad@crys.ras.ru (V.A.); 3 Smart Engines Service LLC, 60-Letiya Oktyabrya Avenue, 9, 117312 Moscow, Russia; ingacheva@gmail.com (A.I.); chukalinamarina@gmail.com (M.C.); 4 Institute for Information Transmission Problems (Kharkevich Institute) RAS, Bolshoy Karetny Lane, 19, 127051 Moscow, Russia

**Keywords:** X-ray imaging, tomography, K-edge subtraction imaging, reference tomography, crystal analyzer, spectral tomography, distortion correction algorithm

## Abstract

To solve the problems of spectral tomography, an X-ray optical scheme was proposed, using a crystal analyzer in Laue geometry between the sample and the detector, which allowed for the selection of predetermined pairs of wavelengths from the incident polychromatic radiation to obtain projection images. On a laboratory X-ray microtomography setup, an experiment was carried out for the first time where a mixture of micro-granules of sodium chloride NaCl, silver behenate AgC_22_H_43_O_2_, and lithium niobate LiNbO_3_ was used as a test sample to identify their spatial arrangement. The elements were chosen based on the presence of absorption edges in two of the elements in the energy range of the polychromatic spectrum of the probing radiation. The method of projection distortion correction was used to preprocess the obtained projections. To interpret the obtained reconstruction results, the segmentation method based on the analysis of joint histograms was used. This allowed us to identify each of the three substances. To compare the results obtained, additional “reference” tomographic measurements were performed: one in polychromatic and two in monochromatic (MoK_α_-, MoK_β_-lines) modes. It took three times less time for the tomographic experiment with the crystal analyzer, while the reconstruction accuracy was comparable to that of the “reference” tomography.

## 1. Introduction

The method of computed tomography, which appeared in the early 20th century as a hardware non-destructive method of layer-by-layer visualization of the internal structure of objects, has not stopped developing for over a century [1], offering users more and more new hardware and software solutions, which, in addition to imaging, allow the identification of areas of the internal structure of tomographic objects [2,3]. Since the discovery of X-rays, it has become clear that the contrast of X-ray images of an object depends on its elemental composition and density [4]. Materials with similar characteristics, such as soft tissues of the human body, are weakly distinguishable from each other in X-ray images when objects are probed with polychromatic radiation. To “illuminate” the desired areas, contrasting is used by introducing absorbing elements, such as iodine, xenon, strontium, etc., into the corresponding tissues of the object [5]. By examining the difference in X-ray images before and after contrasting, it is possible to understand the structure of the organ of interest. However, to do this, it is necessary to take two images separated in time; taking into account that the object could move during this time, the resulting image left much to be desired. To solve this problem, the technique of K-edge subtraction imaging (KES imaging) was proposed [6,7]. In this case, the source radiation was chosen so that the K-edge of absorption of the element of interest was between the two characteristic lines of the incident radiation; for example, the K-edge of absorption of iodine lies between K_α1_ and K_α2_ of lanthanum, and the K-edge of xenon lies between K_α1_ and K_α2_ of cerium. By filtering the necessary emission line of radiation, two images were obtained in a relatively short time interval, which solved the problem of blurring the difference resulting image. At present, studies are being carried out, the aim of which is to determine effective imaging protocols (the tube current, the filter type, etc.) on laboratory sources in KES modes [8].

The idea of using X-ray projections at different wavelengths to detect elements by K-edge absorption has also found its application in tomography, forming a separate area of research called spectral tomography [9]. Different approaches are used to obtain images in different energy ranges. For example, several consecutive tomographic scans are performed, changing the accelerating source voltage (Dual-energy CT) with each scan [10,11]. In this approach, any change in the sample between individual scans leads to errors in data interpretation. In 2006, tomographs with two X-ray tubes operating in different energy ranges simultaneously appeared [12], and the problem of displacement of the probed object ceased to be relevant. However, the structural design of such devices leaves them in the expensive segment. When recording images with the simultaneous use of several (usually two) sources (Dual-source CT) [13,14,15], it is necessary to use specialized reconstruction software [14].

In addition, schemes with a polychromatic source and subsequent filtration of radiation by specially selected pairs of filters (Ross filter method) to obtain quasimonochromatic projection images with subsequent tomographic processing (Rainbow CT) are proposed [16,17,18,19]. This approach requires a careful selection of materials and filter thicknesses, and the scanning is performed in several stages, which entails difficulties in stabilizing a small object during micro-CT measurements, as in the case of the Dual-energy CT technique.

One possible solution is to use dual-layer detectors (Dual-Layer CT), where the first detecting layer is also a radiation filter so that the upper layer registers and filters out the long-wave part of the spectrum, and the lower layer of the detector receives the filtered short-wave part of the spectrum [20,21,22,23]. This method is based on the assumption that the first layer of the detector absorbs all long-wave radiation, and the short-wave part of the spectrum does not interact with it in any way. In reality, this condition is difficult to observe, which leads to problems in the interpretation of the obtained results. In addition, such detectors are more difficult to manufacture and are not widely used.

With advances in technology, it has become possible to use linear and two-dimensional energy dispersive detectors to solve spectral tomography problems [24,25,26,27,28,29,30,31]. This approach makes it possible to directly obtain multichannel projection images of an object, where each channel corresponds to a particular wavelength range. However, at the moment, the recorded images are low resolution, for example, 80 × 80 pixels (250 μm per pixel) [27] or 480 × 32 pixels (1.125 mm per pixel) [31], which is not sufficient for microtomography tasks. In [32], the results of the comparison of two approaches, Photon-Counting CT and Dual-Layer CT, are presented.

Previously, we proposed an X-ray optical scheme in which a crystal analyzer in Laue geometry is installed after the sample, which allows one to select predetermined pairs of wavelengths from the incident polychromatic radiation to obtain projection images [33,34]. This scheme allows three projections—two monochromatic and one polychromatic—to be registered simultaneously, and all necessary sets of projections for tomographic reconstruction are typed in one scan. In this case, there are no problems related to the change/displacement of the sample between scans, and the use of a single radiation source solves the problem of matching reconstructions for different wavelengths. In addition, such a scheme can be easily implemented in existing tomographs by installing just one optical element, the crystal analyzer. However, it should be noted that monochromatic projection images recorded on the detector are distorted due to the deviation of the projection direction from the incident radiation direction as a result of diffraction on the crystal, and, therefore, data correction at the preprocessing stage of the tomographic reconstruction algorithm is required [33].

This paper describes the first tomographic experiment using a crystal analyzer in the proposed optical scheme for nondestructive testing applications. To obtain spectral projections, the crystal analyzer was placed between the sample and the detector. A mixture of micro-granules of sodium chloride NaCl, silver behenate AgC_22_H_43_O_2_, and lithium niobate LiNbO_3_ was used as a test sample. The potential integration of lithium niobate into silicon photonics [35,36] adds it to the lineup of promising materials today. As a reference experiment, classical tomographic measurements in polychromatic and monochromatic modes were performed, where a single crystal monochromator was used in the optical pathway between the source and the sample. The results of tomography in the polychromatic mode do not allow for a component-by-component separation, while tomography in two modes—at the energy before the absorption edge of one of the components and after the absorption edge—allows for a component-by-component separation of the mixture under study. In the latter experiment, however, it takes approximately 3 times longer to measure. The method of correction of projection distortions arising in the scheme with the crystal analyzer, and the results of its application, are given in the section “Distortion correction algorithm”. The following section presents the results of reconstruction according to the measurements with the crystal analyzer and single crystal monochromator. The segmentation method based on the analysis of joint histograms was used to interpret the obtained results. KES imaging for detecting micro-particles of silver behenate and lithium niobate in sodium chloride is described in detail in the same section. The results obtained are discussed in the Conclusion.

## 2. Materials and Methods

### 2.1. Description of Laboratory Set-Up

**Figure 1:**
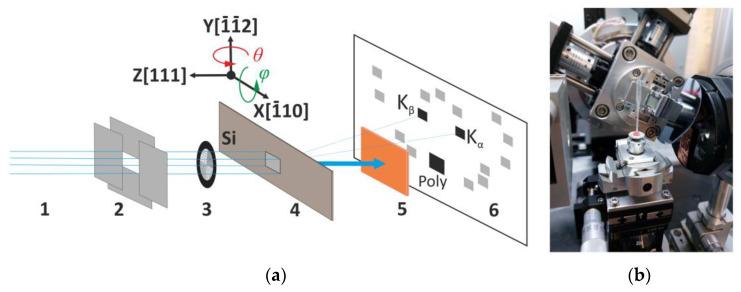
The scheme of the experiment. (**a**) 1—polychromatic X-ray beam; 2—system of slits; 3—study sample (for example, metal grid); 4—crystal analyzer Si(111); 5—filter or absorber of passed beam; 6—CCD-camera. (**b**) Photo of laboratory experimental setup.

Measurements were performed on a laboratory homemade tomographic setup designed and developed in FSRC “Crystallography and photonics” RAS [37,38]. The setup consists of an X-ray source (conventional X-ray tube), multi-axis goniometer, and CCD camera. In subsequent experiments, an X-ray tube with a molybdenum anode and focus 2.0 × 14.0 mm (h × v) was used. Probing conditions: accelerating voltage—40 kV, current—40 mA. The size of the illuminated area was regulated by two mutually perpendicular slits and was 3 × 3 mm. A 330 µm thick silicon single crystal Si(111) was used as an analyzer. The use of a thicker crystal leads to a decrease in the intensities of the diffracted beams, as well as the appearance of a “double” image [39]. Initially, the crystal was mounted so that the plane (111) was perpendicular to the incident beam. Then, by rotating around the X, Y, and Z axes (Figure 1), the analyzer was adjusted to the maximum reflections K_α_ and K_β_ for the {111}-type crystallographic planes. The procedure of crystal analyzer alignment in Laue geometry is described in more detail in [34].

Immediately in front of the crystal on the goniometer, the sample in the study was mounted. Tomographic measurements were performed with an angular step of 1 degree in the rotation range from 0 to 200 degrees around the vertical Y axis. X-ray projection images obtained for the two characteristic lines, as well as those that passed through the “crystal-filter” system, were recorded on a two-dimensional Ximea xiRay11 CCD camera with a sensitive element (pixel) size of 9 μm. To prevent overexposure of the detector by an intense polychromatic beam, a 400 µm copper filter was used. Taking into account the low intensity of the K_β_-line, the exposure time of one frame was 120 s.

The use of a crystal analyzer made it possible to select two characteristic lines of the molybdenum anode—K_α_ and K_β_—from the polychromatic radiation of the standard X-ray tube. As a result of one measurement, three radiographic projections (one polychromatic and two monochromatic) were recorded at once. Let us call this tomographic experiment “spectral” tomography.

For comparison, a series of separate experiments were performed in the traditional scheme, when a single crystal monochromator Si(111) in Bragg geometry was placed in front of the sample to select K_α_ and K_β_. Furthermore, measurements were performed without the single crystal monochromator, i.e., in polychromatic radiation. The operating mode of the X-ray tube (emission spectrum) and the range of rotation angles of the studied sample were chosen the same as in the previous case. The exposure times were 3 s for polychromatic radiation, 60 s for the K_α_-line case, and 120 s for the K_β_-line case. Let us call these three measurements “reference” tomography.

### 2.2. Description of the Sample

For the X-ray tube with a molybdenum anode, the K_α_- and K_β_-lines were 17.479 and 19.608 keV, respectively. Only two chemical elements with K absorption edges are in this energy range: Zr (17.998 keV) and Nb (18.986 keV). Both metals were not investigated in their pure form in this work. As an alternative, micro-granules of lithium niobate crystal (LiNbO_3_), whose absorption edge is close to the absorption jump of pure niobium, were chosen.

**Figure 2:**
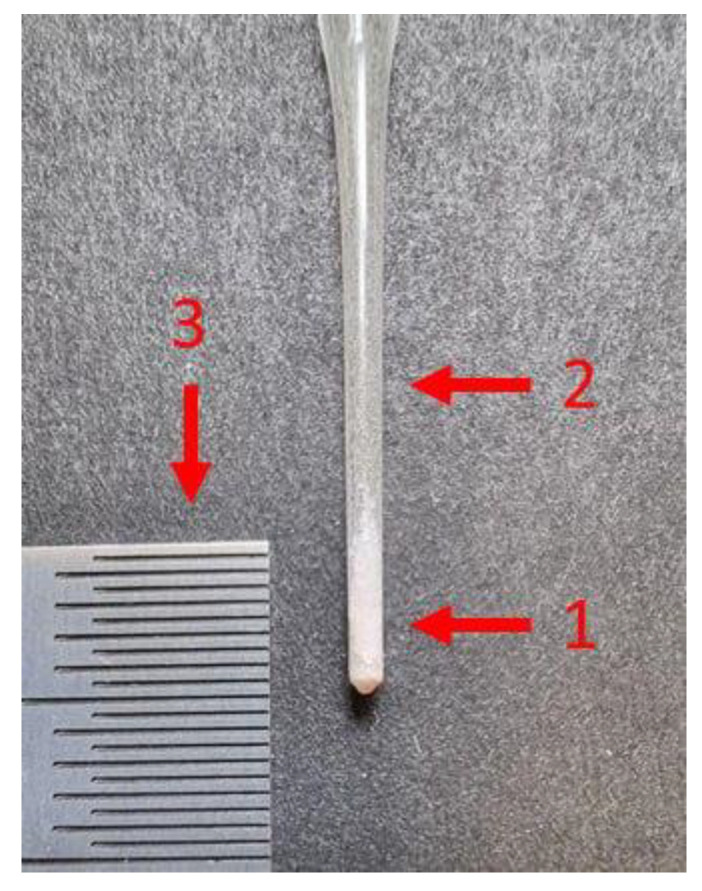
The picture of the sample under study: 1—sample in capillary; 2—empty capillary; 3—ruler with millimeter marks.

To observe the expected effect of spectral tomography on the available equipment, the sample was prepared as follows. At the bottom of the capillary micro-granules of silver behenate (AgC_22_H_43_O_2_), lithium niobate (LiNbO_3_) was successively poured, and sodium chloride (NaCl) was poured last. At the boundaries of the layers, mixtures were formed mechanically. Since the color of the components is the same white, the layers are visually indistinguishable (Figure 2).

**Figure 3:**
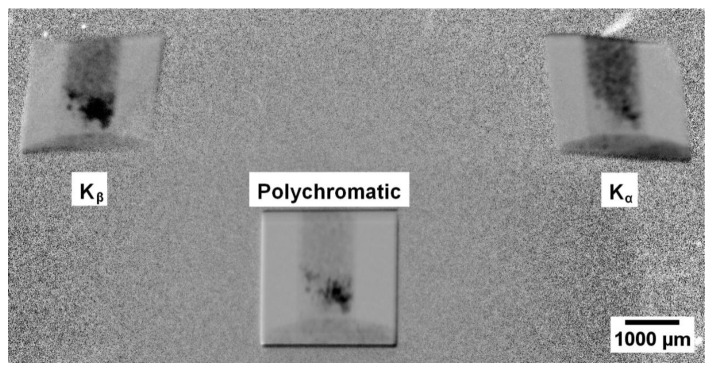
Example of one experimental projection. The images of the studied object obtained for two characteristic lines are visible: K_β_- (**left**) and K_α_-lines (**right**). The image of the polychromatic beam that passed through the object (in the **center**) is also observed.

It should be noted that the NaCl compound has no absorption edges in the polychromatic energy range of the probing spectrum, while for AgC_22_H_43_O_2_, the absorption edge is equal to the value of 25.514 keV of pure silver. The maximum particle size was no more than 100 μm. The mixture thus obtained was placed in a thin capillary with a diameter of 1.15 mm and a wall thickness of 10 μm (stated by the manufacturer). Figure 3 shows an example of projection at the simultaneous fixation of three X-ray beams that passed through the crystal analyzer—two deflected with energies of characteristic lines and passed through the crystal. Since the crystal analyzer was mounted behind the object, the contrast in each of the images is due to the attenuation of the corresponding energy lines by the object material.

**Figure 4:**
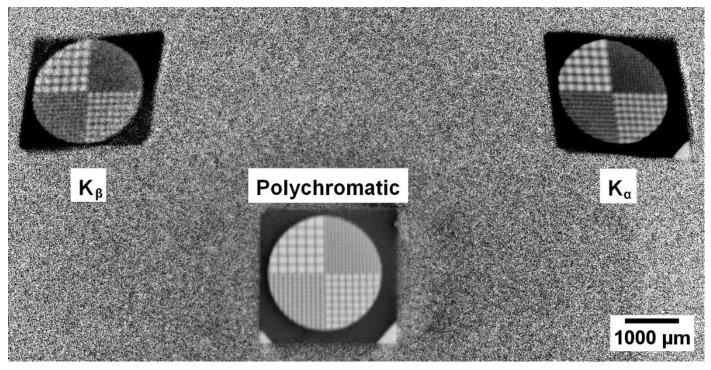
Experimental projection of the gold calibration grid.

To calculate the parameters in the method of correction of geometric distortions arising on the monochromatic projections using the measuring circuit with the crystal analyzer [33], a gold metal grid with a variable period and wall thickness of 20 µm was used as a test object (Figure 4).

### 2.3. Distortion Correction Algorithm

The projection formed by a polychromatic beam passing through the object was recorded without changing the direction of ray propagation and, therefore, without geometric distortions (Figure 4, bottom projection). Standard methods of tomographic image processing [40], including algorithms of tomographic reconstruction, can be applied to the set of such projections. The situation is different with the projections obtained for the Kβ- and Kα-lines (Figure 4, top left and right, respectively); they are registered after the reflection on the crystal analyzer planes and, accordingly, are geometrically distorted. To perform a pixel-by-pixel comparison of the images of reconstructions obtained from three sets of projections, polychromatic and reflections, it is necessary to bring them into the same coordinate system. For this purpose, let us take the coordinate system of the polychromatic projection as the basic one, and for the projections from the reflections, we search for the parameters of the geometric transformation relative to the polychromatic projection. In our optical scheme, straight lines are transformed into straight lines without bending, so the distortion of images is described by the projective transformation [41]. The projective transformation T, in homogeneous coordinates, maps a point (x,y,w) to a point ($x^{\prime },y^{\prime },w^{\prime })$ and is given in the form:

(1) $$\left(\begin{array}{c} {\bar{x}}^{\prime }\\ {\bar{y}}^{\prime }\\ w^{\prime } \end{array}\right)=\left(\begin{array}{c} t_{11}\;\;\;\;t_{12}\;\;\;\;t_{13}\\ t_{21}\;\;\;\;t_{22}\;\;\;\;t_{23}\\ t_{31}\;\;\;\;t_{32}\;\;\;\;t_{33} \end{array}\right)\left(\begin{array}{c} \bar{x}\\ \bar{y}\\ w \end{array}\right)$$

To convert from homogeneous coordinates to ordinary coordinates, we divide the first two coordinates by the third w:

(2) (wx/w,wy/w,w/w)→(x,y,1)

where the number *w* is the scale factor.

In the matrix of coefficients T, the parameter $t_{33}$ will be taken equal to 1 since the value of the parameter is taken out as a multiplier, which is reduced when passing from the homogeneous to the usual coordinates. Thus, 8 degrees of freedom (8 unknown parameters) remain in the matrix. It is possible to find them by considering four points, no three of which lie on the same line. Since each point has two coordinates, the system of linear equations built on 4 points will find 8 unknown parameters. For the system to be nonsingular, no three points must lie on the same line [41].

**Figure 5:**
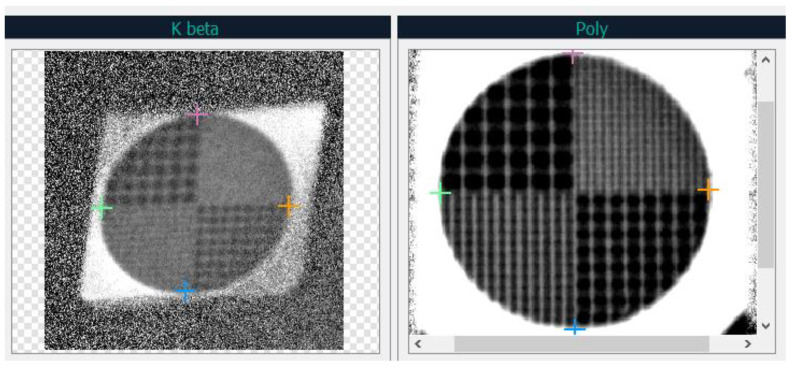
Example of selected characteristic points in the projection from the K_β_ reflex (**left**) and polychromatic projection (**right**). Points on the polychromatic projection (**right**) are reference points. Points on the projection from the K_β_ reflex are points of a projectively distorted quadrilateral, for which it is necessary to find the distortion parameters.

Consider the image of the polychromatic projection and the projection from the K_β_ reflection. Since the problem is to find the transformation parameters at which the points of projections from reflections pass into the corresponding points of the undistorted polychromatic projection, it is sufficient to select 4 points on the images that pass into each other to find the distortion parameters. To simplify the search for such points, a test object was taken, a gold metal grid, which has characteristic points—in the same corners of the grid are the intersections of lines. An example of selected characteristic points at the corners of the grid is shown in Figure 5. The points that must cross into each other are highlighted with crosses of the same color (pink, orange, blue, green).

A system of linear equations of 8 equations with 8 unknowns is made from the selected points. To ensure that the system is not singular, the condition of not lying on the same line of three points must be satisfied. The system is solved using the Gaussian method (sequential elimination of unknowns). Solving the system gives a direct transformation, i.e., a transformation for the transition from a normal image to a distorted one. Applying the inverse transformation matrix to the projection images from the reflections gives the aligned images. To calculate the inverse transformation, the search for the inverse matrix is performed by the Gauss–Jordan elimination method [42].

**Figure 6:**
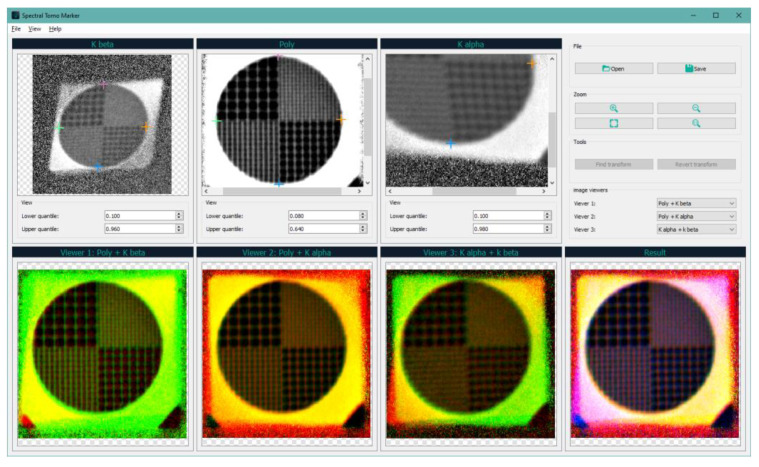
The main window of the program is used to search for projective distortion parameters.

The program “Spectral Tomo Marker” was written to find the parameters of projective transformation, the main window of which is shown in Figure 6.

The program allows you to load a frame with a test sample, from which three areas corresponding to three projections are cut out (the top line of images in the program). Then, the user selects 4 points on each of the three projections. The parameters of two projective transformations (for the projections from the characteristic lines) are calculated on the selected points and then applied to the areas of the original test frame with projections. To check the correctness of the proposed algorithm, the method of constructing a three-channel image is used, which allows visual determination of whether the proposed approach to geometric distortion compensation is correct (bottom line of images in the program). Then, the console version of the program is run, which processes all the frames of the sample experiment with the found parameters. After all registered projections are processed, three-dimensional reconstruction of the object is performed by one of the classical algorithms of tomographic reconstruction.

## 3. Results and Discussion

**Figure 7:**
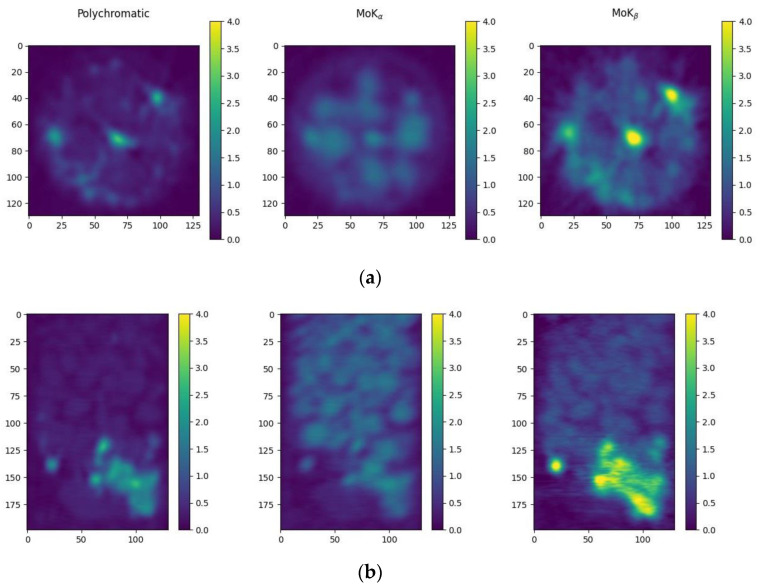
Horizontal (**a**) and vertical (**b**) cross sections of the reconstructed images of the sample obtained in the experiment with the crystal analyzer (**left**—reconstruction from the projections in the polychromatic beam, **center**—from the projections in the MoK_α_-radiation, **right**—in the MoK_β_-radiation).

After applying a distortion correction algorithm to the projections obtained in the crystal analyzer scheme (Figure 1), a reconstruction was performed (Figure 7) [43,44]. The crystal analyzer installed behind the sample highlighted three energy ranges. On the left, Figure 7 shows the reconstruction from the projections in the polychromatic beam transmitted through the crystal analyzer. The center is from the projections in the MoK_α_ radiation highlighted by the crystal analyzer, and the right is the MoK_β_-radiation. Horizontal (Figure 7a) and vertical (Figure 7b) cross-sections of the reconstructed images after Gaussian filtering to remove noise are depicted.

**Figure 8:**
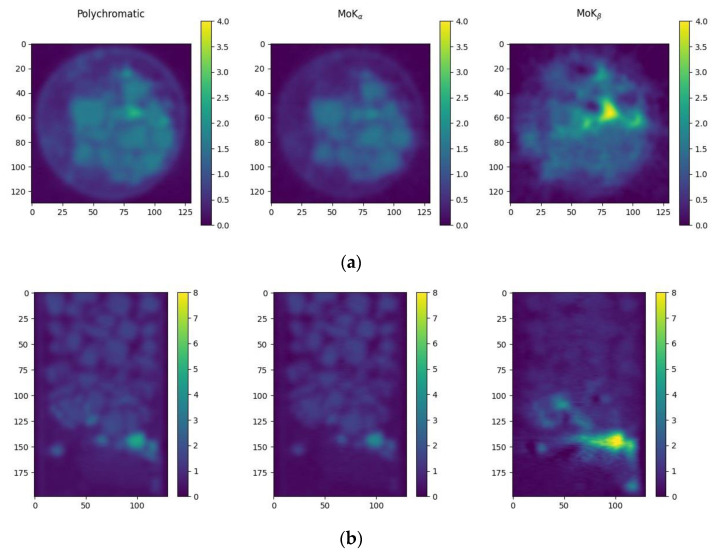
Horizontal (**a**) and vertical (**b**) cross sections of the reconstructed images of the sample obtained in three separate measurements (**left**—reconstruction from the projections in the polychromatic beam without a monochromator, **center**—from the projections in the MoK_α_ reflection of the monochromator, **right**—in the MoK_β_ reflection of the monochromator).

Figure 8 shows the results of tomographic reconstruction from the projections obtained in the tomographic scheme with a single crystal monochromator. Three tomographic measurements were performed. In the first, the sample was probed with a wide polychromatic spectrum. In the second, the sample was probed with MoK_α_ energy radiation. In the third, MoK_β_-line radiation was used. The lines were cut by a single crystal monochromator placed between the source and the sample, i.e., in the second and third cases, monochromatic radiation fell on the sample. The images were reconstructed using three separate tomographic measurements: in the polychromatic spectrum and at two energies corresponding to the characteristic MoK_α_ and MoK_β_-lines. Figure 8 shows horizontal (a) and vertical (b) cross-sections of the reconstructed images of the sample. On the left is the reconstruction from the projections in the polychromatic beam (without the monochromator), in the center are the projections in the MoK_α_ reflection of the monochromator, and on the right is the MoK_β_ reflection of the monochromator.

The cross sections (horizontal and vertical) of the reconstructions in Figure 7 and Figure 8 obtained from the projections in the polychromatic mode do not provide information on the spatial distribution of the mixture components. However, analysis of the images obtained in different spectral ranges makes it possible to identify the location of lithium niobate because the niobium absorption edge lies between the MoK_α_ and MoK_β_ energies.

Note that visually the results of reconstruction from the “spectral” and “reference” tomography projections are quite similar. However, the time spent on measurements in the scheme with the crystal analyzer is approximately three times less than the time spent on the “reference” tomography.

When probing with monochromatic radiation, the problem of qualitative interpretation of the results is trivial in the case when each voxel of the volume contains a single material. The absorption curves for the materials are tabulated in a wide range of energies, and the value of the absorption coefficient can be related to the composition of the local volume. If, however, a mixture of several components whose density may vary is present in a local volume, the linear absorption coefficient of the mixture turns into a linear combination of components, and the approximation of the contributions of each component may be coarse since there is only one equation and several unknown coefficients describing the contribution of each component to the mixture. In such a case, tomography is performed several times at different wavelengths to obtain additional information about the composition [45].

When using a probing polychromatic spectrum, the “averaged” value of the absorption coefficient is more difficult to interpret [46]. First, the linear combination in terms of the material mixture components is supplemented by a linear combination in terms of the contributions of the energy lines of the probing spectrum. Due to the presence of the so-called “spectrum hardening effect” when passing through the object, these weight coefficients change their values. In this regard, the approximation of the final result becomes less accurate [47,48].

To interpret the obtained quantitative brightness values in voxels of the reconstructed volumes, a parallelepipedic region was chosen, measuring 199 × 80 × 80 (1,273,600 in total) voxels, lying completely inside the sample, without capturing the capillary walls and space around the sample. The mask corresponding to the voids inside the object was determined for the obtained volume. For this purpose, a threshold binarization was applied to the images reconstructed from polychromatic projections using the Otsu method [49]. The voxels of this mask were excluded from further processing and analysis but were not removed from the 3D images. Thus, the dimensions of the resulting images did not change.

**Figure 9:**
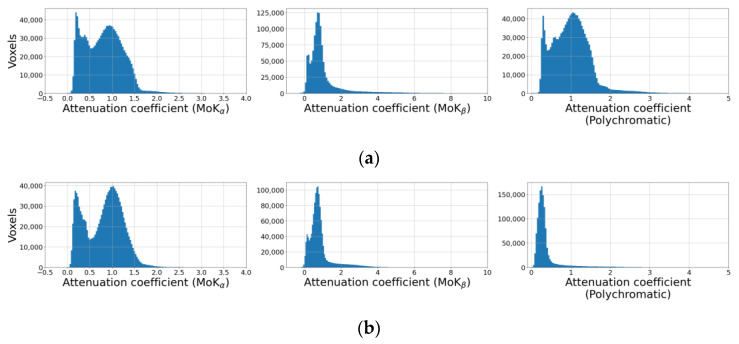
Distribution of the attenuation coefficient for different spectral images: (**a**) classical scheme, (**b**) crystal analyzer scheme.

Histograms of absorption coefficient distributions within selected regions of reconstructed volumes with distant voxel values belonging to the space between crystals are shown in Figure 9. The threshold cutoff does not allow us to distinguish any of the mixture components. Even the averaged absorption coefficients for silver behenate, lithium niobate, and sodium chloride cannot be determined from the histogram.

However, the histograms (Figure 9, right column) clearly show that the distributions of reduced absorption coefficients in polychromatic spectra in the cases of “reference” tomography and tomography with the crystal analyzer are different, despite the same X-ray source used. The difference in spectra is explained by the following. In the “reference” tomography experiment, a spectrum attenuated by the sample was recorded. In the experiment with the crystal analyzer, on the other hand, the registered spectrum does not contain the brightly expressed (reflected by the crystal) MoK_α_ and MoK_β_ spectral lines, parts of the spectrum absorbed by the silicon crystal analyzer, 330 µm thick, or the copper filter, 400 µm thick. The filter was installed in front of the detector window to realize the simultaneous registration of transmitted X-rays, which were deflected by the crystal analyzer. Thus, the polychromatic beam in the “reference” scheme contained relatively more long-wavelength radiation, which resulted in a higher effective value of the reconstructed attenuation coefficient.

**Figure 10:**
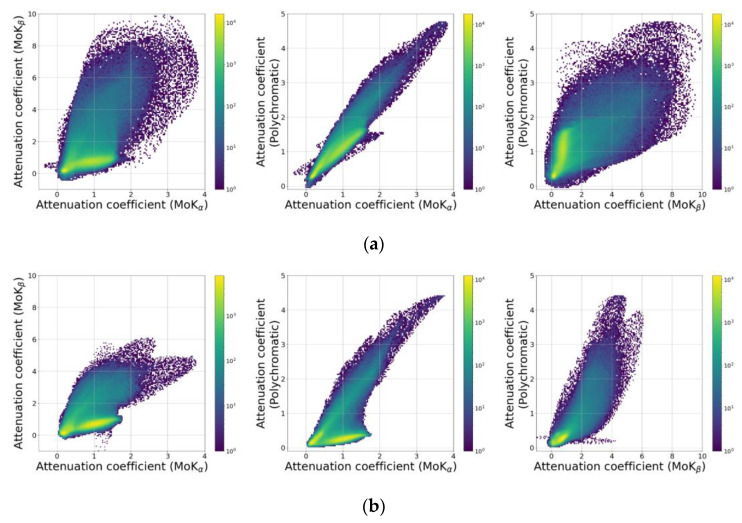
Joint histograms of the absorption coefficient distributions in the 3D reconstructed image sections for the three separate “reference” tomography experiments (**a**) and for the crystal analyzer experiment (**b**). The **left** column is the distribution of the absorption coefficient values for MoK_α_ and MoK_β_. In the **center**, MoK_α_ and polychromatic beam; on the **right**, MoK_β_ and polychromatic beam.

To interpret the obtained reconstruction results, the joint histograms of the absorption coefficient values distribution were plotted (Figure 10). The histograms for the “reference” tomography case are shown in Figure 10a, and the crystal analyzer case in Figure 10b. Since to measure the projections of the “reference” tomography (when measuring the projections in monochromatic modes) the sample had to be removed during the adjustment of the MoK_α_ and MoK_β_ reflections of the crystals, the reconstructed 3D images of the object appeared displaced relative to each other. Their alignment, which is necessary to obtain joint histograms, was performed manually.

**Figure 11:**
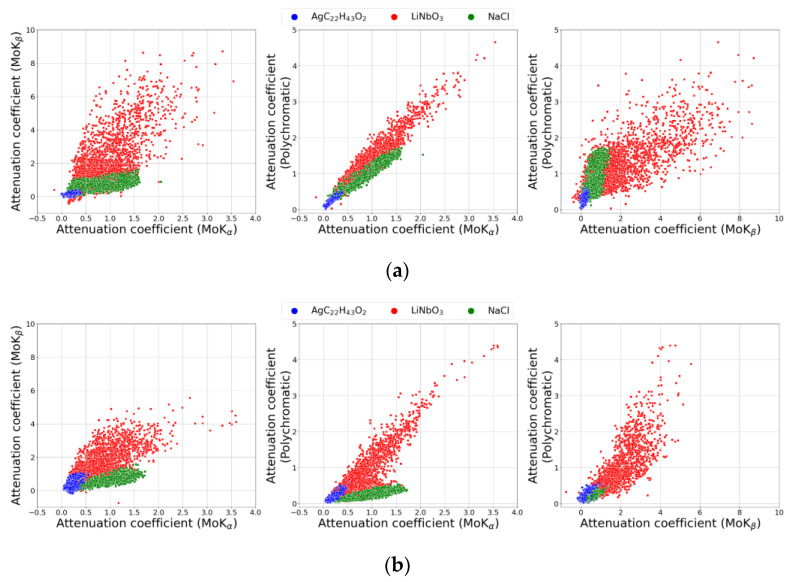
Voxel clustering result of 3D reconstructed image sections: (**a**) for three separate “reference” tomography experiments, (**b**) for crystal analyzer measurements. The **left** column is the distribution of absorption coefficient values for MoK_α_ and MoK_β_. In the **center**, MoK_α_ and polychromatic beam; on the **right**, MoK_β_ and polychromatic beam.

Three regions corresponding to AgC_22_H_43_O_2_, LiNbO_3_, and NaCl are already observed in the joint histograms. To identify the boundaries of these areas and classify the voxels according to their belonging to a particular substance, a clustering procedure was carried out using the Variational Bayesian estimation of a Gaussian mixture algorithm implemented in the Scikit-learn software library [50]. An illustration of the clustering results is shown in Figure 11, where each dot corresponds to one voxel of the sample volume, and the color of the dot identifies the substance to which the voxel belongs. To improve readability, the graphs show only 10,000 randomly selected points out of 1,273,600.

**Figure 12:**
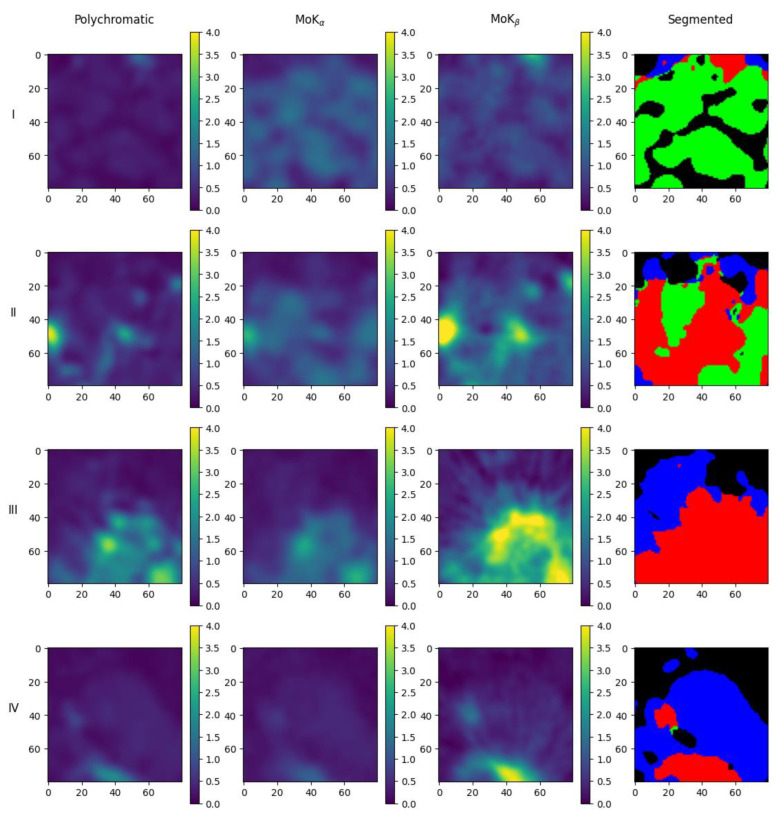
Images of horizontal slices of the object obtained in the experiment with the crystal analyzer (first column—polychromatic radiation, second—MoK_α_, third—MoK_β_), and the corresponding distribution map of substances (last column): green—NaCl, red—LiNbO_3_, blue—AgC_22_H_43_O_2_. Row **I** is the 100th layer on the z-axis (counting from top to bottom), **II** is the 125th layer, **III** is the 150th layer, **IV** is the 175th layer.

**Figure 13:**
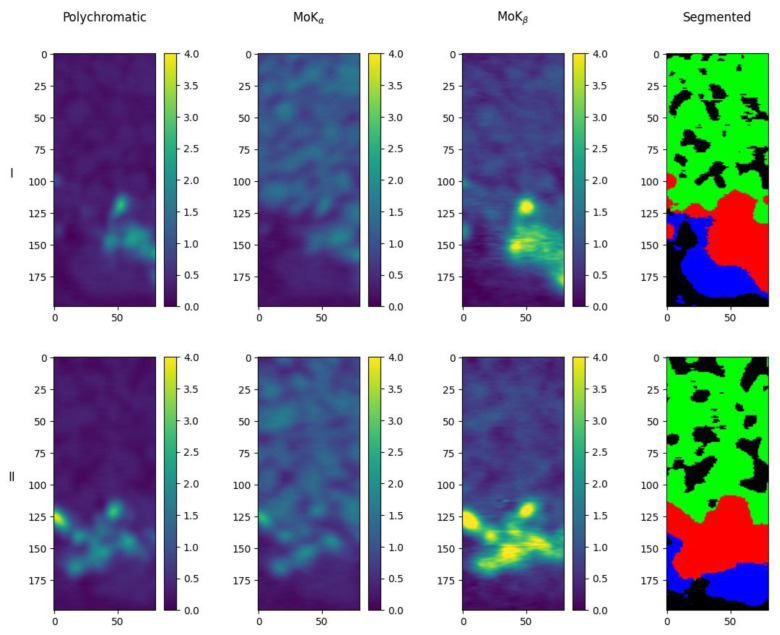
Images of two mutually perpendicular vertical slices of the object obtained in the experiment with the crystal analyzer (first column—polychromatic radiation, second—MoK_α_, third—MoK_β_) and the corresponding distribution map of substances (last column): green—NaCl, red—LiNbO_3_, blue—AgC_22_H_43_O_2_. Row **I** is the 50th layer on the x-axis, **II** is the 50th layer on the y-axis.

By analyzing the reconstruction results for the two performed tomographic measurements (with the crystal analyzer and “reference” tomography), it can be concluded that the obtained estimates of the mean values for NaCl and AgC_22_H_43_O_2_ are within the measurement error, and the estimate for LiNbO_3_ is underestimated. This can be explained by the fact that the size of some of the granules used in the mixture was smaller than the spatial resolution of the method. The local volume could simultaneously contain sodium chloride and lithium niobate, silver behenate and lithium niobate, or the niobate crystal occupied a smaller volume than the local voxel volume. Using the results of clustering, we can transfer the information about which voxel corresponds to which substance to the three-dimensional image of the object under study. Figure 12 and Figure 13 show horizontal and vertical slices of the object with the initial images obtained in the MoK_α_-, MoK_β_- lines and in polychromatic radiation, and the corresponding map of substances distribution in the sample for the experiment with the crystal analyzer. The maps look similar to the images obtained for the “reference” tomography. One can see that the silver behenate is concentrated at the bottom of the object, there is an area containing lithium niobate above it, and the upper half is filled with sodium chloride, which corresponds to the filling of the capillary with the materials under study (see Section 2.2).

## 4. Conclusions

This work is the first experimental demonstration of the capabilities of spectral tomography with a crystal analyzer. The use of the crystal analyzer allowed for the separation of two characteristic lines of the molybdenum anode, specifically *K_α_* and *K_β_*, from the mixed radiation emitted by a standard X-ray tube. This resulted in the recording of three distinct X-ray projections or images.

The tomography does not use an energy-dispersive detector, which allows the collection of projections using a conventional CCD. The sample does not receive the additional radiation load that occurs with dual-energy or dual-source tomography technology.

Three additional “reference” tomographic measurements, one in polychromatic and two in monochromatic (MoK_α_-, MoK_β_-lines) modes, were performed to compare the results obtained. It took three times less time to achieve reconstruction accuracy in the crystal analyzer tomography experiment comparable to that of the “reference” tomography. In such measurements, most of the operations fall on the software part of the tomographic method.

The test object was a capillary with silver behenate (AgC_22_H_43_O_2_), lithium niobate (LiNbO_3_), and sodium chloride (NaCl). After the correction of geometric distortions of the projections collected in the crystal analyzer circuit, tomographic reconstruction was performed. Joint histograms were plotted for the selected areas of the reconstructed 3D digital images. Analysis of the joint histograms allowed segmentation of the reconstructed images, which allowed identification of each of the three regions.

## Data Availability

The data presented in this study are available on request from the corresponding author. The data are not publicly available because the research results are part of a dissertation work that has not been defended. After the defense of the scientific qualification work, the data can be published in the public domain.
